# Effectiveness of Prehabilitation Modalities on Postoperative Outcomes Following Colorectal Cancer Surgery: A Systematic Review of Randomised Controlled Trials

**DOI:** 10.1245/s10434-024-15593-2

**Published:** 2024-06-24

**Authors:** Daniel Steffens, Finley Nott, Cherry Koh, Wilson Jiang, Nicholas Hirst, Ruby Cole, Sascha Karunaratne, Malcolm A. West, Sandy Jack, Michael J. Solomon

**Affiliations:** 1 Surgical Outcomes Research Centre (SOuRCe), Royal Prince Alfred Hospital, Sydney, NSW Australia; 2 Faculty of Medicine and Health, Central Clinical School, The University of Sydney, Sydney, NSW Australia; 3 Institute of Academic Surgery (IAS), Royal Prince Alfred Hospital, Sydney, NSW Australia; 4 Department of Colorectal Surgery, Royal Prince Alfred Hospital, Sydney, NSW Australia; 5 Cancer Sciences, Faculty of Medicine, University of Southampton, Southampton, UK; 6 National Institute for Health and Social Care Research, Southampton Biomedical Research Centre, Perioperative and Critical Care Theme, University Hospitals Southampton, Southampton, UK

**Keywords:** Colorectal cancer, Surgery, Prehabilitation, Postoperative complication, Systematic review, Meta-analysis

## Abstract

**Background:**

Postoperative morbidity in patients undergoing curative colorectal cancer surgery is high. Prehabilitation has been suggested to reduce postoperative morbidity, however its effectiveness is still lacking.

**Objective:**

The aim of this study was to investigate the effectiveness of prehabilitation in reducing postoperative morbidity and length of hospital stay in patients undergoing colorectal cancer surgery.

**Methods:**

A comprehensive electronic search was conducted in the CINAHL, Cochrane Library, Medline, PsychINFO, AMED, and Embase databases from inception to April 2023. Randomised controlled trials testing the effectiveness of prehabilitation, including exercise, nutrition, and/or psychological interventions, compared with usual care in patients undergoing colorectal cancer surgery were included. Two independent review authors extracted relevant information and assessed the risk of bias. Random-effect meta-analyses were used to pool outcomes, and the quality of evidence was assessed using Grading of Recommendations, Assessment, Development, and Evaluations (GRADE) guidelines.

**Results:**

A total of 23 trials were identified (*N* = 2475 patients), including multimodal (3 trials), exercise (3 trials), nutrition (16 trials), and psychological (1 trial) prehabilitation. There was moderate-quality evidence that preoperative nutrition significantly reduced postoperative infectious complications (relative risk 0.65, 95% confidence interval [CI] 0.45–0.94) and low-quality evidence on reducing the length of hospital stay (mean difference 0.87, 95% CI 0.17–1.58) compared with control. A single trial demonstrated an effect of multimodal prehabilitation on postoperative complication.

**Conclusion:**

Nutrition prehabilitation was effective in reducing infectious complications and length of hospital stay. Whether other multimodal, exercise, and psychological prehabilitation modalities improve postoperative outcomes after colorectal cancer surgery is uncertain as the current quality of evidence is low.

**Protocol Registration:**

Open Science Framework (https://doi.org/10.17605/OSF.IO/VW72N).

Globally, the incidence of colorectal cancer is growing. Over the lifespan, approximately 1 in 23 men and 1 in 25 women will develop colorectal cancer.^1^ Despite this, if detected early, surgery alone or in combination with chemotherapy or radiotherapy can provide excellent survival outcomes.^2^ However, colorectal cancer surgery carries significant postoperative morbidity, consequently increasing the length of hospital stay, slowing recovery and increasing health care costs.^3^ Therefore, colorectal cancer treatment is associated with a significant burden on patients and the healthcare system. There is a need to reduce morbidity in this population.

Recently, preoperative modifiable risk factors, including poor physical, nutritional and psychological aspects, have been associated with increased risk of postoperative morbidity.^4–7^ This has resulted in the development of many prehabilitation randomised controlled trials aimed at optimising preoperative patient health in an attempt to reduce postoperative morbidity. Recent randomised trials have focused on unimodal or multimodal interventions, including exercise, nutrition and/or psychological support. In other cancers, there is strong evidence suggesting that prehabilitation is effective in reducing postoperative complications and length of hospital stay.^8,9^

In colorectal cancer, previous systematic reviews have focused on specific populations (e.g., frail patients), introduced high risk of bias with the inclusion of non-randomised trials, included trials with active controls (i.e., rehabilitation after surgery), explored the effectiveness of single preoperative interventions only (i.e., exercise), did not follow recommendations on appraising and synthesising the evidence and/or are outdated.^8,10–13^ In addition, two major randomised trials have been published in the last 12 months (i.e., PHYSSURG-C and PREHAB).^14,15^ Thus, further analysis is warranted.

This study aimed to systematically review the effectiveness of prehabilitation modalities on reducing postoperative morbidity and length of hospital stay in patients undergoing colorectal cancer surgery. Improved understanding on the effectiveness of prehabilitation interventions will provide better recommendations for the management of colorectal cancer patients, future prehabilitation guidelines and on the development of future research.

## Methods

### Protocol and Registration

This review followed the Preferred Reporting Items for Systematic Reviews and Meta-Analyses (PRISMA) statement, and methods recommended by the Cochrane Handbook for Systematic Reviews of Interventions.^16,17^ The review protocol was registered on the Open Science Framework platform (https://osf.io/dashboard; 10.17605/OSF.IO/VW72N).

### Study Selection

Studies meeting the following eligibility criteria were included: (1) randomised controlled trials describing the effectiveness of prehabilitation (including exercise, nutrition and/or psychological interventions) in patients undergoing colorectal cancer surgery, when compared with control (i.e., usual care, minimal intervention, or an active intervention not affecting the outcomes of interest [e.g., delivered 30-days postoperatively]); and (2) reported postoperative complications and/or length of hospital stay outcomes. Trials reporting on mixed populations (e.g., >5% of patients not having colorectal cancer) and studies published as abstracts from conference proceedings were excluded.

### Data Sources and Searchers

A comprehensive search strategy was developed with the support of an experienced librarian from the University of Sydney. The search included a combination of text words and Medical Subject Headings for ‘randomised controlled trials’ AND ‘preoperative’ AND ‘cancer’ AND ‘prehabilitation’ (including ‘exercise’ OR ‘nutrition’ OR ‘psychological’ interventions) AND ‘postoperative outcomes’ (including ‘complications’ or ‘length of hospital stay’). Citation tracking of the included trials and previous literature reviews were also conducted. The search was employed in the CINAHL (Ovid), Cochrane Library, Medline (Ovid), PsycINFO (Ovid), AMED (Ovid) and Embase (Ovid) databases in April 2023.

Two review authors (DS and FN or WJ) independently screened study titles, abstracts and full text of all identified studies using the Covidence systematic review software (www.covidence.org). Any disagreements between the review authors were resolved by discussion with a third author (MS or CK).

### Data Extraction and Risk-of-*Bias* Assessment

Two independent review authors (DS and FN or WJ) performed data extraction using a standardised data extraction sheet. Any disagreements between the review authors were resolved by discussion with a third author (MS or CK). Data extracted included study characteristics, details of prehabilitation intervention and control groups, and outcomes of interest. Data reported as median (and interquartile range, 95% confidence interval [CI], range or *p*-value) were converted to mean and standard deviation using the recommendation strategies of the Cochrane Handbook.^17^ When appropriate, for trials presenting three arms (e.g., two active interventions and one control), the two active interventions were combined.

Risk of bias was assessed using the revised Cochrane risk-of-bias tool for randomised controlled trials (RoB 2).^18^ Two review authors (DS and FN or WJ) independently assessed risk of bias for all included trials. Disagreements between the review authors were resolved by discussion with a third author (MS or CK). Overall risk of bias was judged as ‘low risk’, ‘some concerns’, or ‘high risk’ of bias.

### Data Synthesis and Analysis

Postoperative complication rates were reported as the number of patients presenting with at least one complication and were used to calculate the pooled treatment effect (relative risk and 95% CIs). Relative risk < 1 favoured prehabilitation interventions. Length of hospital stay was reported as mean and standard deviation and was used to calculate the pooled treatment effect (mean difference and 95% CIs). Positive mean differences favoured prehabilitation interventions. Data were pooled using random effects meta-analysis when there was acceptable homogeneity across outcomes and prehabilitation interventions. All meta-analyses were performed using Comprehensive Meta-Analysis software (Biostat Inc., Englewood, NJ, USA). When data could not be included in a meta-analysis, descriptive summary tables were performed.

The quality of evidence for each outcome was evaluate using the Grading of Recommendations, Assessment, Development, and Evaluations (GRADE) approach and rated as ‘high quality of evidence’, ‘moderate quality of evidence’, ‘low quality of evidence’ or ‘very low quality of evidence’.^19^ The quality of evidence was downgraded by one level accordingly to the following criteria: (1) risk of bias (≥25% of included trials presenting one or more domains classified as high risk of bias); (2) inconsistency (statistically significant heterogeneity [I^2^ >50%] or ≤75% of trials with findings in the same direction); (3) imprecision (dichotomous outcomes with sample size <300 participants, or for continuous outcomes with sample size <400 participants); and (4) publication bias (publication bias identified by visual inspection of funnel plots if >10 trials were included). The indirectness criterion was not considered as we only included the colorectal cancer population with relevant outcomes and direct comparisons. For single trials with <400 participants, inconsistency and imprecision (i.e., sparse data) were downgraded and rated as ‘low quality’ evidence. The quality of the evidence could be further downgraded to ‘very low quality’ of evidence if risk-of-bias limitations were found.

## Results

### Study Selection

**Figure 1:**
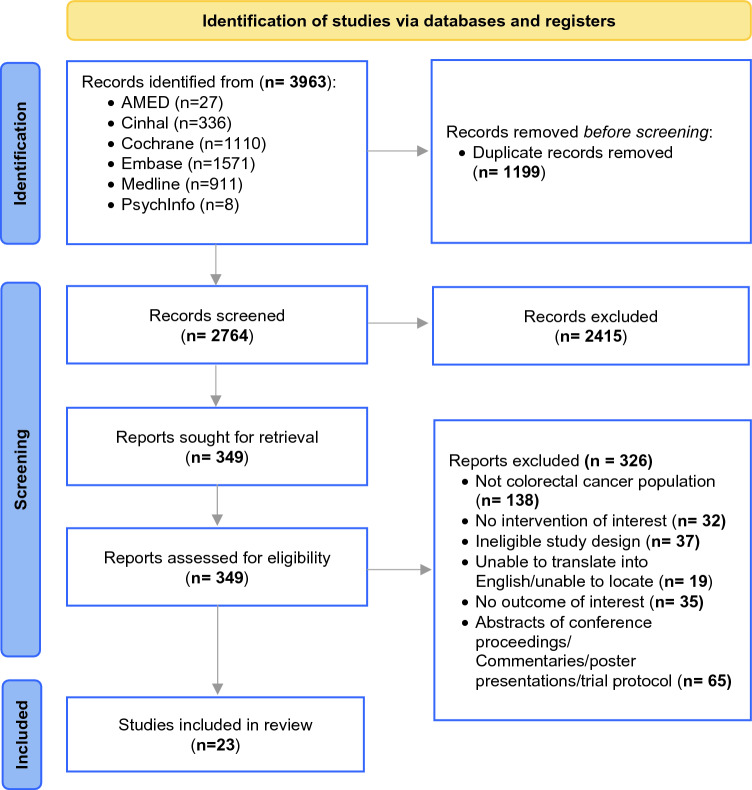
PRISMA flow diagram

Of the 3963 studies identified in the initial search, 23 trials (including 2475 patients) met the eligibility criteria and were included (Fig. 1).^14,15,20–41^

### Study Characteristics

The identified trials investigated the effectiveness of multimodal (exercise, nutrition and psychological interventions) and unimodal prehabilitation (exercise, or nutrition or psychological interventions). The sample size of the included trials ranged from 20 to 668 (average = 105 participants). Only eight trials had a sample of ≥ 100 participants.^14,15,20–23,28,37,38^ The average age of the included participants was 66 years, and the duration of the intervention for multimodal, exercise, nutrition and psychological trials was 4 weeks, 6 weeks, 1 week and 1 h, respectively. Detailed information on the included trials can be found in Table 1.

**Table 1:** Characteristics of the included trials

Author, year	Characteristics	Intervention I	Intervention II	Control	Outcomes
Braga, 2002^20^	Mean age, years (SD): 62.6 (9.3) Sex, female: 41 (41%) Sample size: 100	Treatment name: Oral immunonutrition (including calories and proteins) [*n* = 50] Description: Liquid diet (1 L/day) supplemented with arginine (12.5 g/L) and n-3 fatty acids (3.3 g/L) (oral impact; Novartis, Bern, Switzerland). Provider: Not specified Mode of delivery: Oral Location: Home Number of times: Daily (1 L) Duration: 5 days Intensity: Not applicable Tailored: No Adherence: Not specified	Treatment name: Oral nutrition supplementation (including calories and proteins) [*n* = 50] Description: Isonitrogenous, isoenergetic specially formulated liquid diet Provider: Not specified Mode of delivery: Oral Location: Not specified Number of times: Daily (1 L) Duration: 5 days Intensity: Not applicable Tailored: No Adherence: Not specified	Treatment name: Standard of care (*n* = 50) Description: No artificial diet before surgery Provider: Not applicable Mode of delivery: Not applicable Location: Not applicable Number of times: Not applicable Duration: 5 days Intensity: Not applicable Tailored: Not applicable Adherence: Not applicable	Complications: Anastomotic leak, non-infectious complications, infectious complications LOS: Length of postoperative stay
Koet, 2021^27^	Mean age, years (SD): 71.5 (10.0) Sex, female: 28 (37%) Sample size: 75	Treatment name: Psychological (*n* = 36) Description: Psychological education (coping strategies and practical, social and relational problems were addressed); Education (colorectal cancer education) Provider: Nurse Mode of delivery: Face-to-face Location: Clinic Number of times: Once Duration: 1 h Intensity: Not applicable Tailored: No Adherence: Not applicable	Not applicable	Treatment name: Standard of care (*n* = 39) Description: Standard of care (combined results of the prior diagnostic investigations are discussed, resulting in a therapeutic proposal and overview of potential alternatives) Provider: Nurse and surgeon Mode of delivery: Face-to-face Location: Clinic Number of times: Once Duration: 20 min Intensity: Not applicable Tailored: No Adherence: Not applicable	Complications: Any complication and major complication according to the Clavien–Dindo classification LOS: Length of postoperative stay
Campillo, 2017^30^	Mean age, years (SD): 69.9 (11.0) Sex, female: 25 (30%) Sample size: 84	Treatment name: Oral immunonutrition (including calories and proteins) [*n* = 42] Description: Immunonutrition (impact oral) Provider: Not reported Mode of delivery: Oral Location: Home Number of times: 3 bottles (237 mL) per day Duration: 8 days Intensity: Not applicable Tailored: No Adherence: Not reported	Not applicable	Treatment name: Standard of care (*n* = 42) Description: Routine preoperative management (normal diet) Provider: Not applicable Mode of delivery: Not applicable Location: Not applicable Number of times: Not applicable Duration: 8 days Intensity: Not applicable Tailored: Not applicable Adherence: Not applicable	Complications: Infectious, major complications, anastomotic leak LOS: Length of postoperative stay
Moriya, 2014^31^	Mean age, years (SD): 64.4 (2.2) Sex, female: 33 (39%) Sample size: 85	Treatment name: Oral high immunonutrition (including calories and proteins) [*n* = 26] Description: 750 mL/day omega-3 fatty acids and RNA (IMPACT) and regular diet Provider: Not reported Mode of delivery: Oral Location: Home Number of times: Daily Duration: 5 days Intensity: Not applicable Tailored: No Adherence: Excellent	Treatment name: Oral low immunonutrition (including calories and proteins) [*n* = 30] Description: 250 mL/day omega-3 fatty acids and RNA (IMPACT) and regular diet Provider: Not reported Mode of delivery: Oral Location: Home Number of times: Daily Duration: 5 days Intensity: Not applicable Tailored: No Adherence: Excellent	Treatment name: Standard to care (*n *= 29) Description: Regular diet Provider: Not reported Mode of delivery: Oral Location: Home Number of times: Daily Duration: 5 days Intensity: Not applicable Tailored: No Adherence: Not applicable	Complications: Anastomotic leak, infectious complications, non-infectious complication, wound infection, ileus LOS: Length of postoperative stay
Moug, 2019^32^	Mean age, years (SD): 46.0 (2.0) Sex, female: 17 (35%) Sample size: 40	Treatment name: Aerobic exercise (*n* = 18) Description: Exercise counselling and walking programme Provider: Not reported Mode of delivery: Unsupervised Location: Home Number of times: Daily Duration: Minimum of 13 weeks Intensity: Participants to increase their average daily step count by 3000 accumulated above their baseline Tailored: Yes Adherence: 75%	Not applicable	Treatment name: Standard of care (*n* = 22) Description: Standard of care Provider: Not applicable Mode of delivery: Not applicable Location: Not applicable Number of times: Not applicable Duration: Not applicable Intensity: Not applicable Tailored: Not applicable Adherence: Not applicable	Complications: Any postoperative complication according to the Clavien–Dindo classification LOS: Length of postoperative stay
Polakowski, 2019^33^	Mean age, years (SD): 59.9 (6.5) Sex, female: 43 (47%) Sample size: 73	Treatment name: Oral nutrition supplementation (probiotic) [*n* = 36] Description: Simbioflora (6 g of fructooligosaccharide, and the probiotics Lactobacillus acidophilus NCFM, L. rhamnosus HN001, L. casei LPC-37, and Bifidobacterium lactis HN019 in the concentration of 10^9^ Provider: Not reported Mode of delivery: Oral Location: Home Number of times: Twice daily (diluted envelope in 100 mL of water) Duration: 7 days Intensity: Not applicable Tailored: No Adherence: Not reported	Not applicable	Treatment name: Placebo nutrition (glucose) [*n* = 37] Description: Maltodextrin (obtained from cornstarch) Provider: Not reported Mode of delivery: Oral Location: Home Number of times: Twice daily (diluted envelope in 100 mL of water) Duration: 7 days Intensity: Not applicable Tailored: No Adherence: Not reported	Complications: Infections and non-infectious complications LOS: Length of postoperative stay
Wierdak, 2021^39^	Mean age, years (SD): 69.2 (9.4) Sex, female: 14 (54%) Sample size: 26	Treatment name: Oral immunonutrition (including calories and proteins) [*n* = 14] Description: Immunonutrition (IMPACT). Provider: Not reported Mode of delivery: Oral Location: Home Number of times: 2 Impact oral/day Duration: 2 weeks Intensity: Not applicable Tailored: No Adherence: Not reported	Not applicable	Treatment name: Oral nutrition supplementation (including calories and proteins) [*n* = 12] Description: Standard protein (Nutridrink) Provider: Not reported Mode of delivery: Oral Location: Home Number of times: 3 Nutridrink protein/day Duration: 2 weeks Intensity: Not applicable Tailored: No Adherence: Not reported	Complications: Any complication LOS: Length of postoperative stay
Zelic, 2012^40^	Mean age, years (SD): Not reported Sex, female: 16 (40%) Sample size: 40	Treatment name: Oral nutrition supplementation (carbohydrate loading) [*n* = 20] Description: Carbohydrate-rich beverage (12.5 g/100 mL carbohydrate, 12% monosaccharide, 12% disaccharides, 76% polysaccharides, 285 mOsmol/kg, Nutricia) Provider: Not reported Mode of delivery: Oral Location: Hospital Number of times: Twice (800 mL at night and 400 mL in the morning of the operation) Duration: 1 day Intensity: Not applicable Tailored: No Adherence: Not reported		Treatment name: Standard of care [*n* = 20] Description: Standard of care (nothing by mouth from the evening prior to operation) Provider: Not applicable Mode of delivery: Not applicable Location: Not applicable Number of times: Not applicable Duration: 1 day Intensity: Not applicable Tailored: Not applicable Adherence: Not applicable	Complications: Any complication
Horvat, 2010^25^	Mean age, years (SD): 62.3 (11.0) Sex, Female: 32 (47%) Sample size: 68	Treatment name: Oral nutrition supplementation (synbiotics) [*n* = 20] Description: Multistrain/ multifiber Synbiotic 2000 (10^10^ of Pediacoccus pentosaceus 5–33:3, 10^10^ of Leuconostoc mesenteroides 32–77:1, 10^10^ of Lactobacillus paracasei subsp. paracasei 19, and 10^10^ of Lactobacillus plantarum 2362. Each dose contains a total of 40 billion lactobacilli plus 10 g of bioactive plant fibres, 2.5 g betaglucan, 2.5 g inulin, 2.5 g pectin, 2.5 g resistant starch) Provider: Not reported Mode of delivery: Oral Location: Not reported Number of times: Twice daily (100 mL) Duration: 3 days Intensity: Not applicable Tailored: No Adherence: Not reported	Treatment name: Oral nutrition supplementation (pre/probiotics) [*n* = 28] Description: Multistrain/ multifiber Synbiotic 2000 (10^10^ of Pediacoccus pentosaceus 5–33:3, 10^10^ of Leuconostoc mesenteroides 32–77:1, 10^10^ of Lactobacillus paracasei subsp. paracasei 19, and 10^10^ of Lactobacillus plantarum 2362. Each dose contains a total of 40 billion lactobacilli plus 10 g of bioactive plant fibres – 2.5 g beta glucan, 2.5 g inulin, 2.5 g pectin, 2.5 g resistant starch). Lactobacilli was heat-inactivated Provider: Not reported Mode of delivery: Oral Location: Not reported Number of times: Twice daily (100 mL) Duration: 3 days Intensity: Not applicable Tailored: No Adherence: Not reported	Treatment name: Standard of care (*n* = 20) Description: X-Prep (Mundipharma) Provider: Not reported Mode of delivery: Oral Location: Not reported Number of times: Not reported Duration: Not reported Intensity: Not applicable Tailored: No Adherence: Not applicable	Complications: Any complication LOS: Length of postoperative stay
Hamamoto, 2018^24^	Mean age, years (SD): 68.9 (9.2) Sex, female: 31 (48%) Sample size: 64	Treatment name: Oral nutrition supplementation (carbohydrate loading) [*n* = 31] Description: Arginaid Water (carbohydrate-rich beverage) Provider: Not reported Mode of delivery: Oral Location: Not reported Number of times: 500 mL Arginaid Water the night before surgery and 250 ml Arginaid Water 2 h prior to induction of anesthesia Duration: 1 day Intensity: Not applicable Tailored: No Adherence: Not reported	Not applicable	Treatment name: Standard of care (*n* = 33) Description: Standard of care, including no restriction to clear water 2 h prior to induction of anaesthesia Provider: Not reported Mode of delivery: Oral Location: Not reported Number of times: Daily Duration: 1 day Intensity: Not applicable Tailored: No Adherence: Not applicable	Complications: Surgical site infection, anastomotic leak and ileus LOS: Length of postoperative stay
Reis, 2019^34^	Mean age, years (SD): 64.3 (12.4) Sex, female: 17 (52%) Sample size: 33	Treatment name: Oral nutrition supplementation (carbohydrate loading) [*n* = 15] Description: Maltodextrin Provider: Not reported Mode of delivery: Oral Location: Not reported Number of times: One dose of Maltodextrin at 6:00am on the morning of surgery, and another dose at 10:00am – 2 h before the time scheduled for the procedure. Duration: 1 day Intensity: Not applicable Tailored: No Adherence: Not reported	Not applicable	Treatment name: Standard of care (*n* = 18) Description: Standard of care (remained in absolute fast since the night before surgery) Provider: Not applicable Mode of delivery: Not applicable Location: Not applicable Number of times: Not applicable Duration: 1 day Intensity: Not applicable Tailored: Not applicable Adherence: Not applicable	Complications: Any complication, major complication, and surgical site infection according to the Clavien–Dindo classification LOS: Length of postoperative stay
Burden, 2017^21^	Mean age, years (SD): 69.8 (11.6) Sex, female: 34 (34%) Sample size: 100	Treatment name: Oral nutrition supplementation (including calories and proteins) and dietary advice (*n *= 55) Description: Nutritional supplements (Fortisip Compact: 10.1 KJ and 0.096 g protein per mL) and dietary advice (leaflet) Provider: Nutritionist Mode of delivery: Oral Location: Not reported Number of times: 250 mL/day Duration: Minimum of 5 days Intensity: Not applicable Tailored: No Adherence: 74%	Not applicable	Treatment name: Dietary advice (*n* = 45) Description: Dietary advice only (leaflet) Provider: Nutritionist Mode of delivery: Oral Location: Not applicable Number of times: Not applicable Duration: Minimum of 5 days Intensity: Not applicable Tailored: Not applicable Adherence: Not applicable	Complications: Any complication, pneumonia, urinary infection and surgical site infection LOS: Length of postoperative stay
Burden, 2011^22^	Mean age, years (SD): 64.9 (9.6) Sex, female: 44 (38%) Sample size: 116	Treatment name: Oral nutrition supplementation (including calories and proteins) and dietary advice (*n* = 54) Description: Supplement (milk-based supplements including 630 kJ and 6 g protein per 100 mL; Fortisip) and dietary advice (leaflet) Provider: Not reported Mode of delivery: Oral Location: Not reported Number of times: Daily (400 mL) Duration: Minimum of 10 days Intensity: Not applicable Tailored: No Adherence: 72%	Not applicable	Treatment name: Dietary advice (*n* = 62) Description: Dietary advice (consisted of increasing energy and protein from food, based on an information leaflet) Provider: Not reported Mode of delivery: Not applicable Location: Not reported Number of times: Daily Duration: Minimum of 10 days Intensity: Not applicable Tailored: No Adherence: Not applicable	Complications: Any complication, pneumonia, infectious complication urinary infection and wound infection
López‑Rodríguez‑Arias, 2021^29^	Mean age, years (SD): 66.5 (9.4) Sex, female: 13 (65%) Sample size: 20	Treatment name: Exercise (not described), oral nutrition supplementation (including calories and proteins), dietary advice, psychological intervention (*n* = 10) Description: Prehabilitation video including physical exercise, nutritional supplementation (high-protein nutritional supplementation, with high vitamin D and CaHMB content (Ensure Plus Advance) with minimum supply of 1.2–1.5 g of protein/kg/day, and relaxation exercises) Provider: Not reported Mode of delivery: Unsupervised Location: Home Number of times: Daily (30–45 min) Duration: 30 days Intensity: Not reported Tailored: No Adherence: Not reported	Not applicable	Treatment name: Standard of care (*n* = 10) Description: Standard of care (participants did not receive any education or recommendation on guidelines for physical activity, nutrition, or relaxation) Provider: Not applicable Mode of delivery: Not applicable Location: Not applicable Number of times: Duration: 30 days Intensity: Not applicable Tailored: Not applicable Adherence: Not applicable	LOS: Length of postoperative stay
Rizvanovic, 2019^35^	Mean age, years (SD): 60.6 (8.5) Sex, female: 23 (46%) Sample size: 50	Treatment name: Oral nutrition supplementation (carbohydrate loading) [*n* = 25] Description: Carbohydrate loading Provider: Not reported Mode of delivery: Oral Location: Not reported Number of times: 400 mL of a clear carbohydrate drink (12.5 g/100 mL maltodextrin, 50 kcal/100 mL, pH 5.0) at 22 h on the evening before surgery and another 200 mL of the carbohydrate drink on the day of surgery, 2 h before anaesthesia induction Duration: 1 day Intensity: Not applicable Tailored: No Adherence: Not reported	Not applicable	Treatment name: Standard of care (*n* = 25) Description: Fasting 8 h before surgery Provider: Not applicable Mode of delivery: Not applicable Location: Not applicable Number of times: Not applicable Duration: 8 h Intensity: Not applicable Tailored: Not applicable Adherence: Not applicable	LOS: Length of postoperative stay
Karlsson, 2019^26^	Mean age, years (SD): 77.8 (8.7) Sex, female: 13 (62%) Sample size: 21	Treatment name: Aerobic, resistance training and respiratory exercise (*n* = 10) Description: Respiratory (inspiratory muscle training – POWERbreathe), functional strength exercises, and aerobic exercise (stair climbing, Nordic walking, and interval walking) Provider: Physiotherapist Mode of delivery: Face-to-face Location: Home (supervised/ unsupervised) Number of times: Supervised (2–3 sessions/week), unsupervised (2–3 sessions/week) Duration: 2–3 weeks Intensity: Inspiratory muscle training (50% of maximal capacity for 30 breaths twice daily, with resistance gradually adjusted to achieve a perceived exertion of 5–7 (Borg scale out of 10), aerobic exercises (perceived exertion of 7–8 (Borg scale out of 10) Tailored: Yes Adherence: 98%	Not applicable	Treatment name: Standard of care (*n* = 11) Description: Standard of care (2-week waiting period with ordinary preoperative information, and advice to follow the recommendation of 150 min/week of moderate physical activity) Provider: Not applicable Mode of delivery: Not applicable Location: Not applicable Number of times: Not applicable Duration: 2–3 weeks Intensity: Not applicable Tailored: Not applicable Adherence: Not applicable	Complications: Any complication, pneumonia, urinary infection, pulmonary embolism and wound infection LOS: Length of postoperative stay
Tesar, 2023^37^ and Tesar, 2022^38^	Mean age, years (SD): 65.3 (11.5) Sex, female: 41 (34.2%) Sample size: 120	Treatment name: Oral nutritional supplements (*n *= 60) Description: Supplement 125 mL (extra 2525 KJ and 24 g of protein). If diabetic, received diabetic ONS 200 mL (provided additional 2520 KJ and 30 g of protein) Provider: Not specified Mode of delivery: Not specified Location: Not specified Number of times: 2× per day Duration: 7 days Intensity: Not applicable Tailored: No Adherence: Not specified	Not applicable	Treatment name: No oral nutritional supplements (*n *= 60) Description: Not specified Provider: Not applicable Mode of delivery: Not applicable Location: Not applicable Number of times: Not applicable Duration: Not applicable Intensity: Not applicable Tailored: Not applicable Adherence: Not applicable	Complications: Any complication and severe complication according to the Clavien–Dindo classification system LOS: Length of postoperative stay
Zhang, 2012^41^	Mean age, years (SD): 64.6 (9.8) Sex, female: 36 (60%) Sample size: 60	Treatment name: Probiotic treatment (*n* = 30) Description: 3 oral bifid triple viable capsules, each containing 0.21 g of B Longum, L. acidophilus and enterocococcus faecalis. Provider: Not specified Mode of delivery: Not specified Location: Not specified Number of times: 3 times per day Duration: 3 days Intensity: Not applicable Tailored: No Adherence: Not specified	Not applicable	Treatment name: Placebo (*n* = 30) Description: Placebo capsules containing maltodextrin Provider: Not specified Mode of delivery: Not specified Location: Not specified Number of times: 3 times per day Duration: 3 days Intensity: Not applicable Tailored: Not specified Adherence: Not specified	Complications: Pneumonia, surgical site infections, infectious complications, anastomotic leak LOS: Length of postoperative stay
Onerup, 2022^14^	Mean age, years (SD): 68.0 (11.0) Sex, female: 268 (40%) Sample size: 668	Treatment name: Aerobic exercise and inspiratory muscle training (*n* = 317) Description: 30 min daily aerobic activity. Inspiratory muscle training 30 × 2 breaths with a threshold device Provider: Physiotherapist Mode of delivery: Variable Location: Not specified Number of times: 30 min daily. Aerobic exercise, inspiratory muscle training twice daily Duration: 14 (±4) days Intensity: Medium (exercise) Tailored: Yes Adherence: 63% (exercise)	Not applicable	Treatment name: Standard of care (*n *= 351) Description: Preoperative mobilisation and breathing exercises Provider: Not specified Mode of delivery: Not specified Location: Not specified Number of times: Not specified Duration: Not specified Intensity: Not specified Tailored: Not specified Adherence: Not specified	Complications: Pneumonia, infectious complication and anastomotic leak according to the Clavien–Dindo classification LOS: length of postoperative stay
Molenaar, 2023^15^	Mean age, years (SD): 68.2 (11.9) Sex, female: 113 (45%) Sample size: 251	Treatment name: Exercise, nutrition and psychological support (*n *= 123) Description: 1 h of aerobic and strength exercises 3 times per week, nutritional intervention to achieve protein target of 1.5 g/kg, multivitamins and vitamin D. Relaxation techniques and deep breathing exercises. Smoking cessation Provider: Dietitian, physicians, kinesiologists or physiotherapists and psychology-trained personnel Mode of delivery: Face-to-face Location: In-hospital Number of times: 3 times per week Duration: 4 weeks Intensity: High (aerobic exercise) Tailored: Yes Adherence: 77.2% (exercise)	Not applicable	Treatment name: Standard of care (*n *= 128) Description: ERAS pathway Provider: Not specified Mode of delivery: Not specified Location: Not specified Number of times: Not specified Duration: Not specified Intensity: Not specified Tailored: Not specified Adherence: Not specified	Complications: Any complications, ileus LOS: Length of postoperative stay
Lee, 2023^28^	Mean age, years (SD): 65.3 (10.5) Sex, female: 55 (34%) Sample size: 161	Treatment name: Immunonutrition (*n *= 79) Description: 400 mL/day immune-nutrient-enriched oral nutrition supplementation. Contains high protein, arginine and omega-3 fatty acids Provider: Newcare Omega, Daesang Life Science, South Korea Mode of delivery: Not specified Location: Not specified Number of times: Not specified Duration: 7 days Intensity: Not applicable Tailored: No Adherence: Not specified	Not applicable	Treatment name: Control (*n *= 82) Description: Normal diet Provider: Not applicable Mode of delivery: Not applicable Location: Not applicable Number of times: Not applicable Duration: Not applicable Intensity: Not applicable Tailored: Not applicable Adherence: Not applicable	Complications: Any complications, infectious complications, non-infections, wound infection, urinary infection, surgical site infection, ileus and pneumonia LOS: length of postoperative stay
Carli, 2020^23^	Mean age, years (SD): 78.7 (7.3) Sex, female: 58 (53%) Sample size: 110	Treatment name: Exercise, nutrition and psychological intervention (*n* = 55) Description: Exercise (aerobic, resistance and stretching exercises); nutrition (target protein intake was 1.5 g/kg, supplement with protein supplement Immunocal, Immunotec etc., if not met); psychological (personalised coping strategies 3 times per week) Provider: Kinesiologist, dietitian, psychology-trained nurse Mode of delivery: Face-to-face Location: Home and hospital prehabilitation unit Number of times: Supervised session (once per week), daily walking, elastic band training (three times per week); relaxation (three times per week) Duration: 4 weeks Intensity: Moderate Tailored: Yes Adherence: 80% (27%)	Not applicable	Treatment name: Exercise, nutrition and psychological intervention (*n* = 55) Description: Exercise (aerobic, resistance and stretching exercises) Nutrition (target protein intake was 1.5 g/kg, supplement with protein supplement Immunocal, Immunotec, etc., if not met); Psychological (personalised coping strategies 3 times per week) Provider: Kinesiologist, dietitian, psychology-trained nurse Mode of delivery: Face-to-face Location: Home and hospital prehabilitation unit Number of times: Supervised session (once per week), daily walking, elastic band training (three times per week); relaxation (three times per week) Duration: 4 weeks Intensity: Moderate Tailored: Yes Adherence: 30% (33%)	LOS: Length of postoperative stay
Rizvanovic, 2023^36^	Mean age, years (SD): 60.4 (8.0) Sex, female: 27 (45%) Sample size: 60	Treatment name: Carbohydrate loading (*n *= 30) Description: 400 mL of a carbohydrate solution at 22 h the night before surgery, and 200 mL of the same solution 2 h before surgery Provider: Nurse Mode of delivery: Face-to-face Location: Hospital Number of times: Twice Duration: Beginning night before surgery Intensity: Not applicable Tailored: No Adherence: Not specified	Not applicable	Treatment name: Conventional fasting protocol (*n *= 30) Description: Stopped all oral intake beginning the midnight before surgery Provider: Nursing staff Mode of delivery: Not applicable Location: Hospital Number of times: Not applicable Duration: Not applicable Intensity: Not applicable Tailored: No Adherence: Not applicable	Complications: Any complications, anastomotic leak, wound infection, ileus, pneumonia LOS: Length of postoperative stay

*CaHMB* calcium-β-hydroxy-β-methylbutyrate, *SD* standard deviation, *ERAS* Enhanced Recovery After Surgery

### Risk of Bias

Information on risk of bias of the included trials can be found in Table 2. Risk of bias due to ‘deviation from the intended intervention’ was one of the domains with increased high risk of bias, while the domain risk of bias in ‘measurements of the outcomes presented’ had the lowest risk of bias. Overall, all prehabilitation trials presented at least some risk of bias.

**Table 2:** Risk-of-bias summary of the included studies

First author, year	Risk of bias arising from the randomisation process	Risk of bias due to deviations from the intended interventions	Missing outcome data	Risk of bias in measurement of the outcome	Risk of bias in selection of the reported result	Overall risk of bias
Braga, 2002^20^	Low	Low	Low	Low	Some	Some
Burden, 2011^22^	Low	Low	Low	Low	Some	Some
Burden, 2017^21^	Low	Low	Low	Low	Some	Some
Carli, 2020^23^	Low	Low	Low	Low	Some	Some
Hamamoto, 2018^24^	High	Some	Low	Low	Low	High
Horvat, 2010^25^	Low	High	High	Low	Low	High
Karlsson, 2019^26^	High	Low	Low	Low	Low	High
Koet, 2021^27^	Some	Low	Low	Some	Some	Some
Lee, 2023^28^	Low	Some	Low	Low	Low	Some
Lopez-Rodriguez, 2021^29^	High	Some	High	Low	Some	High
Campillo, 2017^30^	High	High	High	Low	Some	High
Molenaar, 2023^15^	Low	Some	Low	Low	Low	Some
Moriya, 2014^31^	Low	Some	Low	Low	Some	Some
Moug, 2019^32^	Some	Some	High	Low	Some	High
Onerup, 2022^14^	High	Low	High	High	Low	High
Polakowski, 2019^33^	Low	High	Low	Low	Some	High
Reis, 2019^34^	Some	Some	Low	Low	Some	Some
Rizvanovic, 2019^35^	Low	Some	Low	Low	Some	Some
Rizvanovic, 2023^36^	Low	Some	Low	Low	Some	Some
Tesar, 2023^37^ and Tesar, 2022^38^	Low	Some	Low	Low	Some	Some
Wierdak, 2021^39^	Some	High	Low	Low	Some	High
Zelic, 2012^40^	Low	High	Low	Low	Some	High
Zhang, 2012^41^	Some	High	Low	Low	Some	High

### Multimodal Interventions

**Fig. 2:**
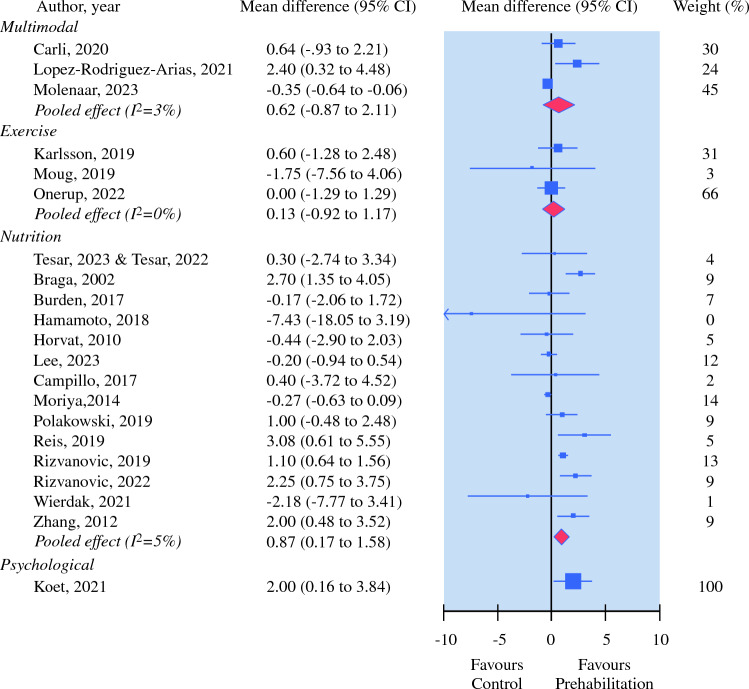
Mean difference for postpoperative length of hospital stay (days) in contrilled trials on efficacy of prehabilitataion for patients undergoing colorectal cancer surgery. Positive values favours prehabilitation interventions.

The effect of multimodal interventions was explored in three trials, including exercise, nutrition, and psychological support (*N* = 381).^15,23,29^ One trial reported the effectiveness of multimodal intervention on postoperative complications (*N* = 251), including any complications, ileus, severe (Charlson Comorbidity Index [CCI] < 20) complications, medical complications, surgical complications, and surgical and medical complications.^15^ No effect of multimodal prehabilitation on postoperative complications (i.e., any complication, ileus, or surgical) was observed (Table 3). However, low quality of evidence of a significant effect favouring multimodal prehabilitation over control was observed on severe (CCI <20) complication rate (relative risk 0.57, 95% CI 0.35–0.92), medical complication (relative risk 0.56, 95% CI 0.34–0.93) and medical and surgical complication (relative risk 0.39, 95% CI 0.16–0.96). Length of hospital stay was reported in three trials (*N* = 381).^15,23,29^ No effect of multimodal prehabilitation on length of hospital stay, when compared with control (mean difference 0.62, 95% CI − 0.87 to 2.11), was observed. The quality of evidence was rated as very low for the length-of-stay outcome (Fig. 2 and Table 3).

**Table 3:** Summary of findings and quality of evidence assessment (GRADE)

Outcomes [first author, year]	Summary of findings		Quality-of-evidence assessment (GRADE)				
	Sample (studies)	Effect size (95% CI)	Risk of bias	Inconsistency	Imprecision	Publication bias	Overall quality of evidence
*Nutrition trials*							
Any complication [Burden, 2011^22^; Burden, 2017^21^; Horvat, 2010^25^; Lee, 2023^28^; Reis, 2019^34^; Rizvanović, 2023^36^; Tesar, 2023^37^; Tesar, 2022^38^; Wierdak, 2021^39^; Zelic, 2012^40^]	724 (9 RCTs)	RR 0.92 (0.73–1.16)	Serious	Serious	Not serious	Undetected	Low
Infectious [Zhang, 2012^41^; Polakowski, 2019^33^; Moriya, 2014^31^; Lee, 2023^28^; Braga, 2002^20^; Burden, 2011^22^; Campillo, 2017^30^]	679 (7 RCTs)	RR 0.65 (0.45–0.94)	Serious	Not serious	Not serious	Undetected	Moderate
Non-infectious [Braga, 2002^20^; Lee, 2023^28^; Moriya, 2014^31^; Polakowski, 2019^33^]	419 (4 RCTs)	RR 0.96 (0.40–2.33)	Serious	Serious	Not serious	Undetected	Low
Anastomotic leak [Campillo, 2017^30^; Braga, 2002^20^; Hamamoto, 2018^24^; Moriya, 2014^31^; Rizvanović, 2023^36^; Zhang, 2012^41^]	453 (6 RCTs)	RR 0.60 (0.30–1.17)	Serious	Not serious	Not serious	Undetected	Moderate
Wound infection [Lee, 2023^28^; Burden, 2011^22^; Moriya, 2014^31^; Rizvanović, 2023^23^]	422 (4 RCTs)	RR 0.57 (0.21–1.56)	Not serious	Serious	Not serious	Undetected	Moderate
Urinary infection [Burden, 2011^22^; Burden, 2017^21^; Lee, 2023^28^; Moriya, 2014^31^]	462 (4 RCTs)	RR 0.88 (0.48–1.61)	Not serious	Serious	Not serious	Undetected	Moderate
Surgical site infection [Burden, 2017^21^; Hamamoto, 2018^24^; Lee, 2023^28^; Reis, 2019^34^; Zhang, 2012^41^]	418 (5 RCTs)	RR 0.57 (0.29–1.11)	Serious	Not serious	Not serious	Undetected	Moderate
Severe complication [Campillo, 2017^30^; Reis, 2019^34^; Tesar, 2023^37^; Tesar, 2022^38^]	237 (3 RCTs)	RR 0.74 (0.36–1.51)	Serious	Serious	Serious	Undetected	Very low
Ileus [Hamamoto, 2018^24^; Lee, 2023^28^; Moriya, 2014^31^; Rizvanović, 2023^36^]	370 (4 RCTs)	RR 0.94 (0.40–2.19)	Serious	Serious	Serious	Undetected	Very low
Pneumonia [Zhang, 2012^41^; Rizvanović, 2023^36^; Lee, 2023^28^; Burden, 2017^21^; Burden, 2011^22^]	497 (5 RCTs)	RR 0.65 (0.32–1.31)	Not serious	Serious	Not serious	Undetected	Moderate
Length of hospital stay (days) [Tesar, 2023^37^; Tesar, 2022^38^; Braga, 2002^20^; Burden, 2017^21^; Hamamoto, 2018^24^; Horvat, 2010^25^; Lee, 2023^28^; Campillo, 2017^30^; Moriya, 2014^31^; Polakowski, 2019^33^; Reis, 2019^34^; Rizvanović, 2019^35^; Rizvanović, 2023^36^; Wierdak, 2021^39^; Zhang, 2012^41^]	1084 (14 RCTs)	MD 0.87 (0.17–1.58)	Serious	Serious	Not serious	Undetected	Low
*Exercise trials*							
Any complication [Karlsson, 2019^26^; Moug, 2019^32^]	61 (2 RCTs)	RR 1.63 (0.67–3.96)	Serious	Not serious	Serious	Undetected	Low
Pneumonia [Karlsson, 2019^26^; Onerup, 2022^14^]	689 (2 RCTs)	RR 0.93 (0.42–2.03)	Serious	Not serious	Not serious	Undetected	Moderate
Wound infection [Karlsson, 2019^26^]	21 (1 RCT)	RR 2.20 (0.23–20.71)	Serious	Serious	Serious	Undetected	Very low
Urinary infection [Karlsson, 2019^26^]	21 (1 RCT)	RR 5.5 (0.29–101.54)	Serious	Serious	Serious	Undetected	Very low
Pulmonary embolism [Karlsson, 2019^26^]	21 (1 RCT)	RR 3.27 (0.14–72.23)	Serious	Serious	Serious	Undetected	Very low
Infectious [Onerup, 2022^14^]	668 (1 RCT)	RR 1.11 (0.90–1.37)	Serious	Not serious	Not serious	Undetected	Moderate
Anastomotic leak [Onerup, 2022^14^]	668 (1 RCT)	RR 1.34 (0.96–1.87)	Serious	Not serious	Not serious	Undetected	Moderate
Length of hospital stay (days) [Karlsson, 2019^26^; Moug, 2019^32^; Onerup, 2022^14^]	729 (3 RCTs)	MD 0.13 (-0.92–1.17)	Serious	Serious	Not serious	Undetected	Low
*Psychological trials*							
Any complication [Koet, 2021^27^]	75 (1 RCT)	RR 0.70 (0.38–1.28)	Not serious	Serious	Serious	Undetected	Low
Severe complication [Koet, 2021^27^]	75 (1 RCT)	RR 0.36 (0.03–3.31)	Not serious	Serious	Serious	Undetected	Low
Length of hospital stay (days) [Koet, 2021^27^]	75 (1 RCT)	MD 2.00 (0.16–3.84)	Not serious	Serious	Serious	Undetected	Low
*Multimodal trials*							
Any complication [Molenaar, 2023^15^]	251 (1 RCT)	RR 0.75 (0.54–1.04)	Not serious	Serious	Serious	Undetected	Low
Ileus [Molenaar, 2023^15^]	251 (1 RCT)	RR 0.71 (0.36–1.37)	Not serious	Serious	Serious	Undetected	Low
Severe (CCI >20) complications [Molenaar, 2023^15^]	251 (1 RCT)	RR 0.57 (0.35–0.92)	Not serious	Serious	Serious	Undetected	Low
Medical complications [Molenaar, 2023^15^]	251 (1 RCT)	RR 0.56 (0.34–0.93)	Not serious	Serious	Serious	Undetected	Low
Surgical complications [Molenaar, 2023^15^]	251 (1 RCT)	RR 0.77 (0.49–1.20)	Not serious	Serious	Serious	Undetected	Low
Medical and surgical complications [Molenaar, 2023^15^]	251 (1 RCT)	RR 0.39 (0.16–0.96)	Not serious	Serious	Serious	Undetected	Low
Length of hospital stay (days) [Carli, 2020^23^; López‑Rodríguez‑Arias, 2021^29^ Molenaar, 2023^15^	381 (3 RCTs)	MD 0.62 (−0.87 to 2.11)	Serious	Serious	Serious	Undetected	Very low

Very low: The true effect is probably markedly different from the estimated effect. Low: The true effect might be markedly different from the estimated effect. Moderate: The authors believe that the true effect is probably close to the estimated effect. High: The authors have a lot of confidence that the true effect is similar to the estimated effect

*CI* confidence interval, *RCT* randomised controlled trials, *RR* relative risk (value <1 favours prehabilitation interventions), *MD* mean difference (positive values favour prehabilitation interventions), *GRADE* Grading of Recommendations, Assessment, Development, and Evaluations, *CCI* Charlson Comorbidity Index

### Exercise Interventions

**Fig. 3:**
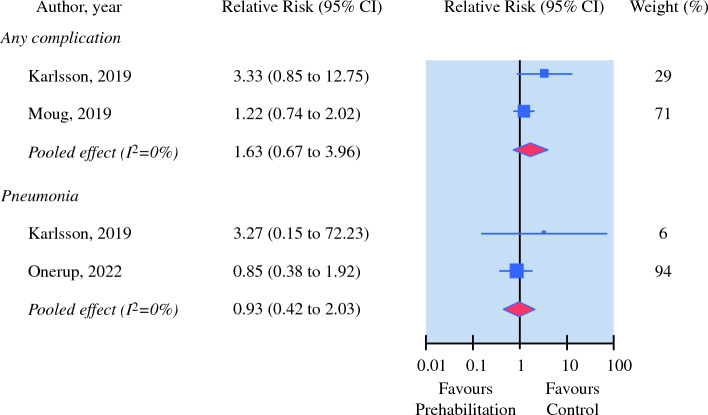
Relative risk for number of postoperative complications in controlled trials on exercise prehabilitataion for patients undergoing colorectal cancer surgery. Values < 1 favours excercise prehabilitation interventions

Three trials investigated the effect of preoperative exercise on postoperative complications and length of hospital stay (*N* = 729).^14,26,32^ Preoperative exercise was not effective on reducing postoperative complications (i.e., any complication, pneumonia, wound infection, urinary infection, pulmonary embolism, infections complications and anastomotic leak) and length of hospital stay. The quality of evidence ranged from moderate to very low for all outcomes reported (Fig. 3 and Table 3).

### Nutritional Interventions

**Fig. 4:**
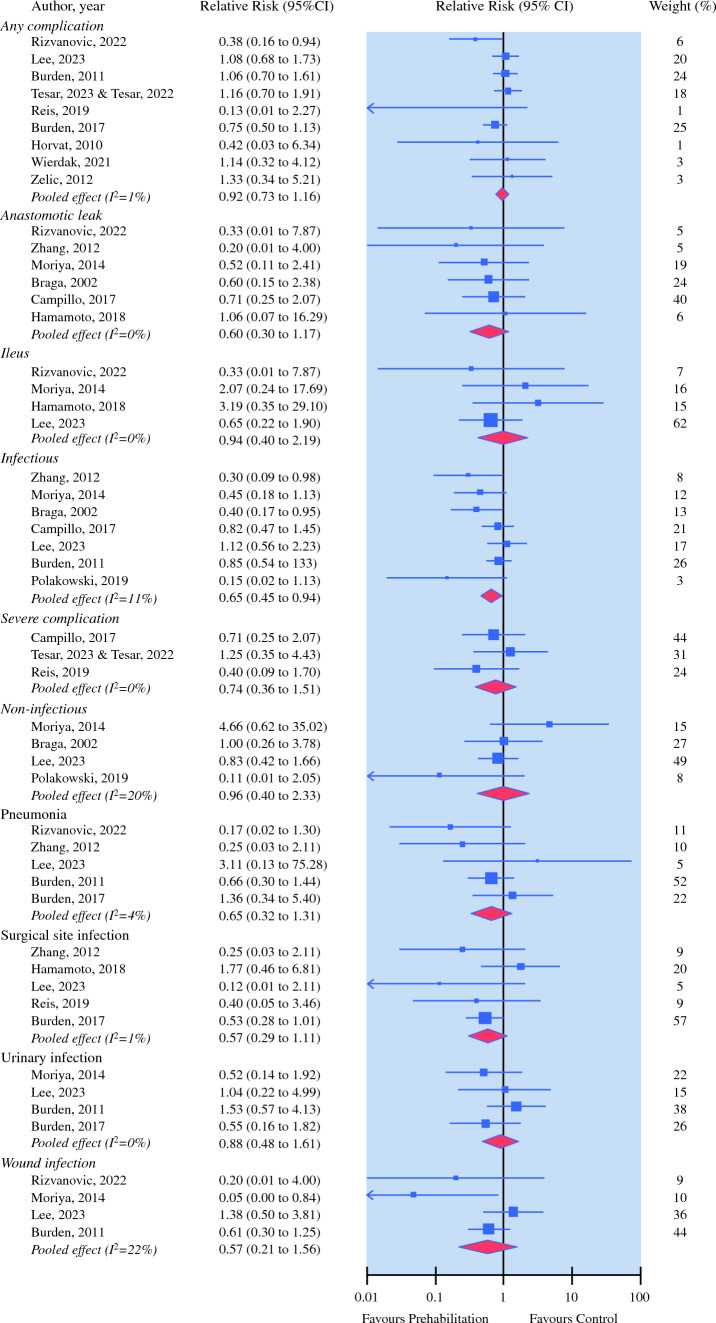
Relative risk for number of postoperative complications in controlled trials on efficacy of nutrition prehabilitataion for patients undergoing colorectal cancer surgery. Values < 1 favours nutrition prehabilitation interventions

A total of 16 trials investigated the effect of preoperative nutrition interventions on postoperative complications and length of stay.^20–22,24,25,28,30,31,33–41^ Pooling estimates from seven trials (*N* = 679) provided moderate quality of evidence of a significant effect favouring preoperative nutrition intervention over control on postoperative infectious complications (relative risk 0.65, 95% CI 0.45–0.94) (Fig. 4).^20,22,28,30,31,33,41^ Preoperative nutrition (14 trials, *N* = 1084) was effective in reducing postoperative length of hospital stay when compared with control (mean difference 0.87, 95% CI 0.17–1.58).^20,21,24,25,28,30,31,33–39,41^ The quality of evidence was rated as low for the length-of-stay outcome. No other significant effect was observed (Fig. 2 and Table 3).

### Psychological Interventions

A single trial investigated the effect of preoperative psychological interventions on complications and length of stay (*N *= 75).^27^ No effect was observed on complications and length of hospital stay. The quality of evidence was low for all outcomes reported (Table 3).

## Discussion

This systematic review and meta-analyses found moderate-quality evidence that preoperative nutrition intervention was effective in reducing infectious complications by 35% and length of hospital stay by approximately 1 day in patients undergoing colorectal cancer surgery. The effect of multimodal, exercise and psychological prehabilitation interventions on postoperative outcomes was uncertain due to the limited number of trials, heterogeneity in reported outcomes, and the low quality of evidence. Currently, there is limited confidence in the effect estimates of prehabilitation following colorectal cancer surgery and the results should be interpreted with caution.

The number of randomised controlled trials investigating the effectiveness of prehabilitation on postoperative outcomes of patients undergoing cancer surgery has increased drastically over the last decade. This has provided a window of opportunity to conduct systematic reviews and meta-analyses on the available evidence. Recently, a number of systematic reviews have been published aimed at synthesising the effects of prehabilitation on preoperative functional capacity, postoperative outcomes and quality of life. A review conducted by Bausys et al. summarised the current evidence on prehabilitation in the management of colorectal cancer patients.^11^ Of the 21 articles identified, 10 were either non-randomised controlled trials or retrospective studies. That review reported that most of the individual studies demonstrated at least some positive effects of prehabilitation on patients’ physical, nutritional, or psychological status and in reducing postoperative morbidity. Interestingly, in the current review, when postoperative outcomes were pooled within different prehabilitation modalities, most of the meta-analyses performed demonstrated no effect. Multimodal prehabilitation trials would expect to have a synergistic effect on outcome improvement, especially when compared with unimodal interventions such as exercise or nutrition alone. The difference between the results of the two reviews may be due to the bias introduced by the inclusion of non-randomised studies in the previous review. This was further evidenced by the systematic review and meta-analysis conducted by Chang et al., where the effect of prehabilitation on frail colorectal cancer patients was described.^10^ The initial significant effect of prehabilitation on postoperative complications (odds ratio 0.51, 95% CI 0.34–0.78) and length of hospital stay (standardised mean difference − 0.34, 95% CI − 0.46 to − 0.23) when randomised and non-randomised studies were included disappeared when only randomised trials were pooled (odds ratio 1.04, 95% CI 0.23–4.64; and standardised mean difference − 0.14, 95% CI − 0.44 to 0.16, respectively).^10^

Previous systematic reviews investigated the effect of other preoperative interventions, including exercise, nutrition, or psychological support. In the review performed by Gillis et al., pooled outcomes of six nutrition prehabilitation studies (including randomised trials and cohort studies) demonstrated a significant reduction in length of hospital stay by almost 3 days when compared with control.^42^ In the review performed by Falz et al. short (< 3 weeks) and long-term (≥ 3 weeks) preoperative exercise interventions had no effect on postoperative complications and length of hospital stay following colorectal cancer surgery.^43^ The evidence from previous psychological prehabilitation reviews is in line with the current findings of this review.^44^ Despite the number of reviews available in the literature, most applied different methodological approaches, including study designs that would introduce high risk of bias within the pooled estimates. In addition, other reviews included active ‘control’ groups (e.g., exercise prehabilitation) that were introduced early in the postoperative period, potentially influencing postoperative outcomes, such as complication and length of hospital stay.^12^

The risk of developing a postoperative complication following colorectal cancer surgery is highest in the first 30 postoperative days.^45^ This is a critical determinant of recovery, long-term outcomes (including quality of life) and treatment costs. Our review found that preoperative nutrition interventions significantly reduced the rates of postoperative infectious complications and length of hospital stay following colorectal cancer surgery. Unfortunately, due to the limited evidence, the effectiveness of other prehabilitation modalities is still lacking. Molenaar et al. conducted a trial investigating the effectiveness of multimodal prehabilitation, and reported a significant effect, when compared with control, on rates of severe complications, medical complications, and combined medical and surgical complications.^15^ Future multimodal trials will allow for data pooling, which will enhance the quality of the current evidence. Within the preoperative nutrition trials, immunonutrition and other oral nutrition supplementations (including carbohydrate loading) were the most tested interventions, however the dosage used varied across most trials. The duration of the nutrition interventions was also inconsistent, with interventions lasting from a couple of hours to a few weeks (7 days on average). Therefore, determining the prehabilitation standard of care for colorectal cancer patients undergoing surgical treatment is somewhat challenging within the current literature. In an attempt to guide future trials, a recent Delphi study identified key research priorities in prehabilitation.^46^ Further recommendations on the development of reporting guidelines, including prehabilitation intervention components, and reporting of core set outcomes are warranted.^47^ Thus, there is a need for the establishment of a core set of outcomes for prehabilitation and the development of prehabilitation guidelines. These steps would enhance the conceptualisation and design of future prehabilitation trials for patients undergoing colorectal cancer surgery. In addition, it is important to acknowledge that the implementation of enhanced recovery after surgery pathways has already led to significant improvements in surgical outcomes, such as reduced length of hospital stay. As a result, it may be more challenging to demonstrate further improvements in complication rates and length of stay when prehabilitation is added to an existing enhanced recovery after surgery program.

Some of the key strengths of this review included the adherence to the Cochrane recommendations; reporting according to the PRISMA statement; inclusion of the latest prehabilitation randomised controlled trials; use of two experienced reviewers to screen studies, extract data and assess risk of bias; use of the Cochrane RoB 2; and use of the GRADE approach to determine the quality of the evidence. Despite this, the current systematic review and meta-analysis has some limitations. Our comprehensive search identified a large number of trials but we may have missed trials stored in the grey literature, therefore publication bias cannot be ruled out. While we pooled trials according to their prehabilitation modalities, the type of intervention, frequency, intensity, duration, mode of delivery, adherence, and progression may vary across the trials. Therefore, identification of the optimal prehabilitation intervention may not be possible. In addition, despite the literature suggesting that prehabilitation should be employed at least 4 weeks before cancer surgery, some of the trials investigated the effectiveness of a single session intervention (e.g., 1 h). Finally, due to the small number of trials identified across each prehabilitation modality, the level of adherence to the interventions was not taken into consideration during the analysis. It is important to note that adherence reporting and definitions are essential for future research and meta-analyses.

The quality of the current prehabilitation literature has been previously described and included deviation from intended interventions, poor outcome reporting and definition, lack of publicly available protocols, underpowered trials, and changes in primary and secondary outcomes.^48^ Thus, there is an urgent need to further understand the barriers and facilitators to the conceptualisation of higher-quality prehabilitation trials. Furthermore, reporting of postoperative outcomes of the identified trials was somewhat heterogenous. While we pooled outcomes describing a specific complication, for some trials the definition of postoperative complications was either slightly different (e.g., using different complication classification systems) or not available (e.g., not reported within the published article or protocol [if available]). This should be taken into consideration when interpreting the results of this systematic review.

## Conclusion

There is moderate quality of evidence that nutrition prehabilitation is effective in reducing infectious complications rates by 35%, and low quality of evidence in reducing length of hospital stay by approximately 1 day in patients undergoing colorectal cancer surgery. The benefit of other prehabilitation modalities, including multimodal, exercise and psychological interventions, is limited due to lack of randomised controlled trials, heterogeneity in reported outcomes, and the low quality of evidence. There are a number of registered prehabilitation randomised controlled trials that may change our confidence in results and effect estimates in the near future.
